# Diabetic Nephropathy: Prescription Trends in Tertiary Care

**DOI:** 10.4103/0250-474X.43007

**Published:** 2008

**Authors:** D. Padmini Devi, Jennifer George

**Affiliations:** Department of Pharmacology, St. John.s Medical College, Bangalore-560 034, India; 1 Department of Pharmacology, KVG Medical College, Sullia-574 239, India

**Keywords:** Diabetic nephropathy, hypertension, prescribing trends, calcium channel blockers

## Abstract

Diabetic nephropathy is a leading cause of end stage renal disease. Drug utilization studies could promote rational drug use. The objective of this study was to evaluate prescribing trends in hospitalized patients with diabetic nephropathy. A prospective, observational study was conducted in a tertiary care hospital. The demographic, disease and treatment data of patients with diabetic nephropathy were collected for a period of six months and analysed. Drugs were classified using World Health Organization recommended Anatomic Therapeutic Chemical classification. A total of 755 drugs (7.4 drugs per prescription) were prescribed to 102 study patients, who were all hypertensive and in late stages of diabetic nephropathy. Drug classes with largest representation were those acting on gastrointestinal tract plus metabolism (37%) and cardiovascular drugs (28%). Calcium channel blockers represented the largest antihypertensive drug class (41%). Almost three-fourths of patients received more than one antihypertensive agent. Approximately 37% of patients did not receive any antidiabetic medication. Of those who did, prescriptions for insulin (91%) exceeded those of oral hypoglycaemic drugs (9%). Antimicrobials accounted for 10.2% of all drugs prescribed, of which 31.8% were quinolones. Drugs prescribed by generic name accounted for 11.98%. While all patients received antihypertensive therapy, more than a third were not on any antidiabetic treatment. Antihypertensive poly-therapy was observed in the majority with calcium channel blockers being most frequently prescribed antihypertensive drug class. Insulin was the preferred to hypoglycaemic drugs.

Diabetic Nephropathy (DN) is a microvascular complication of insulin dependent (IDDM) and non-insulin dependent (NIDDM) diabetes mellitus characterised by persistent proteinuria, decline in glomerular filtration rate (GFR) and increased morbidity and mortality in diabetics^1^. Studies have suggested that the prevalence of DN is approximately 40% in diabetic patients^2^. DN is the most common cause of end stage renal disease (ESRD)^3^. Physicians face challenges in selecting, initiating and individualising appropriate drug therapy for patients with DN.

The study of prescribing patterns is a component of medical audit that monitors and evaluates prescribing practices and recommends necessary modifications to achieve rational drug use^4^. The objective of this study was to analyse current drug prescribing trends in the management of DN in hospitalised patients undergoing tertiary care.

This prospective, cross sectional, observational study was conducted in the inpatient ward of the Nephrology Department of a tertiary care teaching hospital over the six-month period, 1^st^ October 2003 to 31^st^ March 2004. All inpatients with the diagnosis of DN made by the consultant nephrologist, undergoing treatment in the Nephrology unit were included. However, patients who were under day care and those who were admitted only for dialysis were excluded. Patients to be included in the study were identified while accompanying the physicians on morning rounds and the diagnosis was confirmed from the inpatient medical records. The demographic details, necessary clinical data and drug details were collected in a specially designed proforma. This study included only one prescription per patient during that hospital stay.

Drugs were classified into different groups based on Anatomic Therapeutic Chemical (ATC) classification^5^. World Health Organization (WHO) recommends this classification system developed by European Association for Pharmaceutical Market and International group. In the ATC classification system, the drugs are divided in to different groups according to the organ system on which they act and or therapeutic and chemical characteristics. These classifications are from the WHO's collaborating Centre for Drug Statistics methodology. Data on utilization of different classes as well as individual drugs were subjected to descriptive analysis.

Of 637 inpatients admitted in the Nephrology ward during the study period, 102 (16%) were found to have DN. The male to female ratio was 2:1. The mean age for developing DN was 55.1±9.5 years. The mean age among males was 54.5±8.75 years and that for females was 55.7±10.1 years.

One hundred patients were diagnosed with NIDDM while two patients were found to have IDDM. Ninety-five patients (93%) suffered from chronic renal failure being either in stage 4 (overt nephropathy) or in stage 5 (ESRD) of DN. The rest were found to be in the stage of micro-albuminuria (stage 3 of DN). Of known risk factors for the development of DN, male gender (67%), family history of DN (12%), diabetes of more than ten years duration (76%), were observed. Hypertension and diabetic retinopathy was found in all patients (100%). The results of analysis of prescriptions for rationality are presented in Table 1. Drugs were categorised into 14 groups based on first anatomical level of ATC classification^5^ as shown in Table 2.

**TABLE 1:** ANALYSIS OF PRESCRIPTIONS IN DIABETIC NEPHROPATHY

Details of prescription	Number (%)
Total number of prescriptions analysed	102
Total number of drugs prescribed	755
Average number of drugs per prescription	7.4
Number of drugs from WHO essential drug list out of total number of drugs prescribed	401 (53)
Number of drugs prescribed by generic name out of total number of drugs prescribed	90 (12)
Total number of injections out of total number of drugs	242 (32)

**TABLE 2:** DISTRIBUTION OF DRUGS IN DIFFERENT CATEGORIES BASED ON ATC CLASSIFICATION PRESCRIBED IN DIABETIC NEPHROPATHY

Drug class (based on ATC classification)	Number N=755	Percentage
A - Drugs for Gastro-intestinal tract and metabolism	281	37. 2
B - Drugs for treatment of disorders of blood and blood forming organs	70	9.3
C - Drugs for Cardiovascular system	211	27.9
D - Dermatological drugs	4	0.5
G - Drugs for Genitourinary systems and sex hormones	27	3.6
H - Hormones for systemic use except sex hormones	4	0.5
J - Anti-infectious drugs for systemic use	77	10.2
L - Antineoplastic and Immunomodulating agents	3	0.4
M - Drugs for Musculoskeletal systems	5	0.7
N - Drugs acting on Nervous System	14	1.8
P - Drugs against parasites and insecticides	0	0
R - Drugs for Respiratory system	42	5.6
S - Drugs for eye and ear	3	0.4
V - Various others	14	1.9

Based on ATC classification, it was found that drugs acting on gastrointestinal system and metabolism and cardiovascular drugs represented the maximum in patients with diabetic nephropathy.

The most frequently prescribed drug classes in DN were presented in Table 3. Antihypertensives were prescribed to all study patients. Amlodipine (68/74; 91.89%) was the most commonly prescribed calcium channel blocker, while furosemide (49/53; 92.45%) and prazosin (40/41; 97.56%) topped the list of diuretics and alpha-blockers respectively. Twenty eight percent of the prescriptions contained a single antihypertensive drug, while 61% contained two and 11% contained three. Of the total of 102 prescriptions, 49% contained one antimicrobial drug, 9.8% had two drugs and 5.9% had three drugs. Omeprazole (25/70; 35.7%), and pantoprazole (13/70; 18.6%) accounted for more than half the total anti-peptic ulcer prescriptions. Preparations of insulin accounted for the majority of hypoglycemic drugs (91%) as illustrated in Table 2.

**TABLE 3:** PATTERNS OF UTILIZATION OF MAJOR DRUG CLASSES

Drug class	*ATC code	Number	Percentage
Antihypertensive drugs (n=186)			
1. Calcium channel blockers	C08CA	74	40
2. Diuretics	C03CA	53	28
3. Alpha blockers	C02CA	41	22
4. Angiotensin converting enzyme inhibitors	C09AA	7	4
5. Beta blockers	C07AB	5	3
6. Miscellaneous group		6	3
Antiinfective drugs (n=77)			
1. Quinolones	J01MA	28	36
2. Cephalosporins	J01DA	21	27
3. Metronidozole	G01AF01	11	14
4. Miscellaneous group		17	22
Antipeptic ulcer drugs (n=70)			
1. Proton pump inhibitors	A02BC	38	54
2. H2 blockers	A02BA	28	40
3. Miscellaneous group.		4	6
Hypoglycaemic drugs (n=69)			
1. Insulin	A10A	63	91
2. Oral hypoglycaemic drugs	A10B	6	9

* Anatomic-Therapeutic-Chemical classification

The five most frequently prescribed agents were amlodipine (68/755) (9%), calcium carbonate (54/755) (7.2%), furosemide (49/755) (6.5%), prazosin (40/755) (5.3%) and ranitidine 28/755 (3.7%). Analysis of the different routes of administration used for 755 drugs showed that 470 (62%) drugs were administered orally, 181 (24%) by intravenous or intramuscular route, 58 (8%) by subcutaneous route for, 25 (3%) by inhalation and 21 (3%) by other routes.

The results of our study revealed that the percentage of in-patients who were treated in Nephrology ward with the diagnosis of DN was 16% of the total number of hospitalised patients in the Nephrology unit over six months. The demographic results revealed that there was a male preponderance with the mean age above 50 years as similar to a previous Indian study conducted by John *et al*.^6^.

The analysis of clinical data showed that the majority of study patients were type 2 diabetics. Known risk factors seen in the present study included male sex, long duration of diabetes (more than 10 years), family history of DN and hypertension^7^. Hypertension and diabetic retinopathy were the two associated major co-morbid factors of DN seen in 100% of patients. The majority of patients (93%) were in late stage of DN (stage 4 or 5) with chronic renal failure.

The average number of drugs per prescription was 7.4, indicating polypharmacy. In research studies, polypharmacy has been defined as the concomitant use of five or more drugs^8^. Generally, a finding of polypharmacy is an indication that there is scope for review of the existing prescribing trends. However, given the distinctive health problems of this particular study population, it is a moot point whether use of five concomitant drugs can be considered to be polypharmacy. Our study thus highlights the issue of definition of polypharmacy for DN with multiple co-morbid factors. This is of vital importance given that polypharmacy requires justification because of the increased risk of drug interactions and errors of prescribing^9^.

In this study it was found that only 12% of the drugs were prescribed by generic name, showing that prescribing by brand name is the norm. Fifty three percent of the prescribed drugs were from the WHO essential drugs list^10^. Around 32% of the total numbers of drugs were given as injections, which are a commonly overused and costly form of drug therapy.

The results of this study showed that people with DN are in need of a wide spectrum of drug classes under ATC classification. The most commonly prescribed therapeutic classes (based on ATC classification) in DN were drugs for gastrointestinal system and drug metabolism (A) (39.7%), drugs for cardio-vascular system (C) (27.9%) and antiinfectives for systemic use (J) (10.2%). Comparative clinical trials and pharmacoeconomic analysis of different drugs from these classes will be highly beneficial for patients with DN.

Analysis of prescribing pattern of antihypertensives revealed that calcium channel blockers (C08CA) (46.3%) and diuretics (C03CA) (26%) were the most frequently prescribed anti-hypertensive drugs to treat hypertension in DN. ACE inhibitors and alpha-receptor blockers (ARBs) reduce the progression of DN and should be preferred in individuals with diabetes plus microalbuminuria^11^. However, in late stages of DN with chronic renal failure or ESRD, hyperkalemia is more likely to develop when ACE inhibitors are prescribed. Also, unlike CCBs, most of the ACE inhibitors need dose modification in renal failure^12^. Hyperkalemia produced by ARBs is comparable to that produced by ACE inhibitors in the presence of renal insufficiency or diabetes^11^. As the majority (93%) of patients in this study were in late stages of DN with chronic renal failure or ESRD, the choice of CCBs and diuretics is logical.

The fact that 100% of study patients were on antihypertensive medication is an indication of the high prevalence of cardiovascular morbidity in DN^1^. Further, about 72% of patients were prescribed more than one antihypertensive drug. The seventh report of the Joint National Committee (JNC VII) also has reported that combinations of 2 or more drugs are needed to achieve the target blood pressure of 130/80 mm of hg in diabetic hypertensive patients^11^.

Insulin was highly preferred over oral hypoglycaemic drugs. It is well established that intensive glycaemic control reduces the rate and progression of micro-albuminuria in diabetics. However, during the phase of declining renal function, insulin requirement falls since the kidney is a site of insulin degradation^12^ and this could be the reason for the striking number (37%), of such patients not receiving any anti-diabetic agent.

Prescriptions of insulin preparations (91%) were more compared to OHAs. This transfer to insulin treatment could be due to avoidance of oral hypoglycaemic drugs, which can accumulate in uraemia and lead to hypoglycaemia^13^. In this study, about 65% of 102 patients received anti-microbial agents which indicated the high prevalence of infections in patients hospitalised with DN.

Of 755 drugs, the top three most commonly prescribed drugs in this study were amlodipine (9%), calcium carbonate (7.2%) and furosemide (6.5%). The high use of amlodipine and furosemide is unsurprising as all patients had concomitant hypertension. High usage of calcium carbonate is explained by the need for phosphate binders in renal failure. The oral route (62.3%) was the most commonly prescribed route of drug delivery even in hospitalized patients with DN.

This study identified a wide variety of classes of drugs prescribed in DN. This was indicative of the wide spectrum of prevailing morbidity patterns in patients with this chronic disease. Inpatient based prescription audit has a major advantage of minimising the ‘drop-outs’ because patient compliance is ensured. The information on drug prescribing patterns can provide a framework for continuous prescription audit in a hospital setting. This can help the prescribers to improve patient management by rationalising prescribing practices.
